# Prospective study of predictors for anxiety, depression, and somatization in a sample of 1807 cancer patients

**DOI:** 10.1038/s41598-024-53212-y

**Published:** 2024-02-07

**Authors:** Veronica Velasco-Durantez, Patricia Cruz-Castellanos, Raquel Hernandez, Adan Rodriguez-Gonzalez, Ana Fernandez Montes, Alejandro Gallego, Aranzazu Manzano-Fernandez, Elena Sorribes, Marta Zafra, Alberto Carmona-Bayonas, Caterina Calderon, Paula Jiménez-Fonseca

**Affiliations:** 1 Department of Medical Oncology, Hospital Universitario Central de Asturias, ISPA, Avenida Roma sn, 33011 Oviedo, Spain; 2 Universidad de Valladolid, Valladolid, Spain; 3 Department of Medical Oncology, Hospital General Universitario de Ciudad Real, Ciudad Real, Spain; 4 Department of Medical Oncology, Hospital Universitario de Canarias, Tenerife, Spain; 5 Department of Medical Oncology, Complejo Hospitalario Universitario de Ourense, Orense, Spain; 6 Department of Medical Oncology, Clínica Universidad de Navarra, Madrid, Spain; 7 Department of Medical Oncology, Hospital Universitario Clínico San Carlos, Madrid, Spain; 8 Department of Clinical Psychology and Psychobiology, Faculty of Psychology, University of Barcelona, Barcelona, Spain; 9 Department of Hematology and Medical Oncology, Hospital Universitario Morales Meseguer, Murcia, Spain; 10 Department of Hematology and Medical Oncology, Hospital Universitario Morales Meseguer, UMU, IMIB, Murcia, Spain

**Keywords:** Cancer, Psychology

## Abstract

In cancer patients, psychological distress, which encompasses anxiety, depression, and somatization, arises from the complex interplay of emotional and behavioral reactions to the diagnosis and treatment, significantly influencing their functionality and quality of life. The aim was to investigate factors associated with psychological distress in cancer patients. This prospective and multicenter study, conducted by the Spanish Society of Medical Oncology (SEOM), included two cohorts of patients with cancer (localized resected or advanced unresectable). They completed surveys assessing psychological distress (BSI-18) before and after cancer treatment and coping (MINI-MAC) and spirituality (FACIT-sp) prior to therapy. A multivariable logistic regression analysis and a Structural Equation Modeling (SEM) were conducted. Between 2019 and 2022, 1807 patients were evaluated, mostly women (54%), average age 64 years. The most frequent cancers were colorectal (30%), breast (25%) and lung (18%). Men had lower levels of anxiety and depression (OR 0.66, 95% CI 0.52–0.84; OR 0.72, 95% CI 0.56–0.93). Colorectal cancer patients experienced less anxiety (OR 0.63, 95% CI 0.43–0.92), depression (OR 0.55, 95% CI 0.37–0.81), and somatization (OR 0.59, 95% CI 0.42–0.83). Patients with localized cancer and spiritual beliefs had reduced psychological distress, whereas those with anxious preoccupation had higher level. SEM revealed a relationship between psychological distress and coping strategies, emphasizing how baseline anxious preoccupation exacerbates post-treatment distress. This study suggests that age, sex, extension and location of cancer, coping and spirituality influence psychological distress in cancer patients.

## Introduction

Cancer is a growing concern worldwide, with the International Agency for Research on Cancer (IARC) estimating 18.1 million cases in 2020, rising to 28 million in 2040. According to the Spanish Society of Medical Oncology (SEOM), there will be an estimated 279,260 cases in 2023, potentially reaching 341,000 in 2040^1^. Its impact on physical and mental health is significant, and its prognosis is often poor. As such, despite advances in diagnosis and treatment, it remains one of the leading causes of death, with 9.89 million fatalities in 2020, and an estimated 113,000 in Spain. This number is expected to grow to 16 million worldwide and 159,000 in Spain by 2040^1^.

It is widely known that cancer patients are more likely to experience psychological distress, such as anxiety, depressive disorders, and somatization, due to the severity of the disease and its unfavorable prognosis^2^. Recent research, including Wang Y-H et al.'s 2020 meta-analysis, has further emphasized the connection between psychological distress and increased mortality risk and poorer survival outcomes^3^.

Psychological distress in cancer patients is conceptualized as psychological harm that affects an individual's functionality. This condition encompasses various emotional and behavioral aspects, emerging as a result of the diagnosis and treatment of the disease^4^ Haga clic o pulse aquí para escribir texto. Previous research has highlighted the significance of depressive disorders and intense emotional response patterns as key factors in reducing the quality of life of these patients^4^. In this context, it is crucial to recognize how anxiety, depression, and somatization, although distinct in their nature, intertwine in the cancer experience^5^. Anxiety may stem from the uncertainty and fear of the future, while depression could be a reaction to the challenges and changes imposed by the illness. Somatization, on the other hand, reflects how emotional stress can manifest in physical symptoms. This comprehensive understanding is essential to appropriately address the psychological needs of cancer patients, thereby improving their quality of life and their ability to cope with the disease^4,6^

Anxiety is the most widely reported psychological disorder found in up to 38% of cancer patients^2^.Age, gender, and cancer location are believed to be mediators, with higher anxiety levels generally seen in younger patients^7^. An Iranian meta-analysis in 2022 and another meta-analysis conducted in Iran revealed a higher prevalence of both anxiety and depression, as well as depression alone in breast cancer patients, respectively^8,9^. Moreover, depression is a common issue among cancer patients, affecting up to 16%^10^. Its prevalence is likely to vary throughout the course of the disease, as various elements, such as the patient's response to their diagnosis, fear of the appearance of symptoms related to the disease, and the adverse effects of treatments, can contribute to it. Additionally, living with the uncertainty of a recurrence or progression of the cancer, as well as the fear of death, can have a significant impact^10^. Relative to somatic symptoms, it has been observed that cancer patients may experience a plethora of symptoms, both from their cancer treatments and the tumor disease. Common examples include pain, fatigue, anorexia, tiredness, lack of energy, trembling and lethargy. A meta-analysis published in 2020 found an association between female gender and a higher level of fatigue in cancer patients^11^. It is important to note that it can be difficult to distinguish between physical symptoms caused by cancer and those caused by psychological issues, which might lead to misdiagnosis and a delay in providing psychological interventions^12,13^.

Coping is an essential psychological factor for cancer patients, as it can provide a sense of protection and adjustment from the negative effects associated with diagnosis, treatment adverse events, the risk of recurrence, and potential socio-economic and familial repercussions. Adaptive coping strategies, such as displaying a fighting spirit or maintaining a positive attitude, have been shown to facilitate positive psychosocial adaptation to the cancer experience. In contrast, those using a non-adaptive coping style, such as fatalism or anxious preoccupation-based approaches, are more likely to experience anxiety, depression, social isolation, and reduced quality of life^14,15^. In addition, research has demonstrated that those with a greater sense of psychospiritual well-being are better able to handle the terminal illness process^16^.

Spirituality is commonly understood as an individual's search for significance and purpose in life, as well as a connection with intangible aspects of existence and transcendence. Patients may draw from their spiritual beliefs to find strength, hope and meaning in the face of a cancer diagnosis and its associated phases. Factors such as prognostic knowledge, family and social support, autonomy, hope and meaning in life have been identified to contribute to psychospiritual well-being, while emotional distress, anxiety, helplessness, hopelessness, and fear of death are known to detract from it process^16^. Patients facing a chronic, incurable, or terminal illness may begin to question the meaning of life. Studies have suggested a positive relationship between religion/spirituality and mental health in cancer patients^17^. Furthermore, meaning-focused interventions may be beneficial for improving quality of life in those with advanced cancer^18^. In addition, spiritual interventions have been found to reduce anxiety, depression, and hopelessness in cancer patients, leading to physical and psychological benefit^17,18^. As such, it is important for the medical team to consider the patient's spirituality, and to provide interventions if necessary or requested^19^.

With all the above in mind, the aim of this study was to evaluate the sociodemographic, clinical, and psychological factors predictive of anxiety, depression, and somatization in cancer patients. Additionally, the correlation between psychological distress and coping, spirituality and age was also explored. Furthermore, Structural Equation Modeling (SEM) was utilized to investigate the relationship between psychological distress before and after treatment and coping strategies.

## Materials and methods

### Patients and study design

This study, sponsored by the Spanish Society of Medical Oncology (SEOM) Bioethics Section, was prospective, observational, and consecutive in nature. It was approved by the Ethics Committee of each institution, as well as the Spanish Agency of Medicines and Health Products, and all participants signed an informed consent form prior to inclusion.

The participants were those who had a histologically confirmed cancer and were candidates for systemic therapy. Individuals under the age of 18 and those with any serious mental illness that would hinder their understanding of the study, as well as any underlying personal, family, sociological, geographic, and/ or medical condition that might impede their participation, were excluded. Moreover, individuals who had already received any systemic cancer treatment or patients with resected metastatic cancer were also excluded.

The study was based on a series of questionnaires that the medical oncologist provided to the patient during the same visit in which they discussed the potential benefits of adjuvant (for resected localized cancer) or palliative (for unresectable advanced cancer) systemic cancer treatment. These forms were completed by the patient at home prior to the start of treatment and then handed in to the study assistants at the next visit. Each form contained clear instructions and specified that its completion was voluntary and anonymous. Patients with resected localized cancer completed the psychological distress questionnaire at the end of their adjuvant treatment, which was 6 months after starting treatment. Subjects with unresectable advanced cancer completed the questionnaire after their first radiological response evaluation study, which was 2–3 months after their antineoplastic treatment began. This was closer to baseline, as this population has a poorer prognosis and increased risk of premature death.

### Measures and variables

The information was collected and updated by medical oncologists specially trained to meet the requirements of the study. Demographic and clinical data (age, sex, marital status, educational level, employment status, cancer location, and stage, and treatment received) were obtained directly from the patients and their medical records. Cancer location was classified into breast, bronchopulmonary, colon, non-colorectal digestive (encompassing esophagus, stomach, pancreas, biliary tract, liver, and anal canal) and all other cases were categorized as 'other'. Stage I-III cancers that had been resected were categorized as resected localized whereas stage III-IV cancers that were deemed unresectable were classified as unresectable advanced.

The participants finished the questionnaires BSI-18 (psychological distress), MINI-MAC (coping), and FACIT-sp (spirituality). The **Brief Symptom Inventory** (BSI-18) was employed, consisting of 18 items which measure the respondent's overall emotional adjustment or psychological distress over the preceding 7 days^20^, each rated on a five-point Likert scale, from zero (not at all) to four (extremely). Raw scores are converted to T-scores based on gender-specific normative data. Following the clinical case-rule criteria^20^ and using the cut-off values recommended by Derogatis^20^, patients with T-scores of 63 or higher were identified as likely experiencing significant anxiety, depression, or somatization^20^.

Cronbach's alpha for the scale has been reported to range from 0.81 to 0.90 ^20^, and its validity has been established among Spanish-speaking populations^21^. In the current study, it was evaluated both at the start and conclusion of adjuvant treatment (in those with resected localized cancer) or following the first response assessment imaging study (in those with unresectable advanced disease).

The **Mini-Mental Adjustment to Cancer** (Mini-MAC) is a 29-item scale that evaluates the cancer-specific coping strategies of individuals^22^. It classifies four coping strategies: anxious preoccupation, helplessness, positive attitude, and cognitive avoidance. The version adapted for Spanish cancer patients was used in this study^23^. It is important to note that the four-factor structure of the Mini-MAC was initially identified in a prior study by our research group, using a different sample of cancer patients. Each item is rated on a four-point Likert scale from one (definitely does not apply to me) to four (definitely applies to me). The higher the score on a given subscale, the more frequently that coping strategy is employed. The omega coefficients for the Spanish version of the score range from 0.76 to 0.90^23^. In this study, the internal consistency of the scale scores ranges from 0.82 to 0.90.

The **Functional Assessment of Chronic Illness-Spiritual Well-Being Scale** (FACIT-Sp) is a 12-item questionnaire which uses a five-point Likert-type scale, with responses ranging from not at all (0) to very much (4). The scale is divided into three subdomains which assess spiritual well-being (meaning, peace and faith)^24^. The FACIT-sp scores vary from 0 to 48, with higher values indicating a higher spiritual well-being. Internal consistency reliability to the full-scale in the Spanish version was 0.87 ^25^.

### Statistical analysis

Descriptive statistics were employed to analyze demographic data and questionnaire responses. Categorial variables were expressed as percentages, while quantitative variables were reported in terms of mean and standard deviation (SD). Pearson’s correlation was used to determine the level of association between psychological variables and age. Analysis of variance (ANOVAs) were conducted to examine variances in psychological distress (anxiety, depression, and somatization) at the onset of the treatment related to demographic and clinical variables. Multivariate logistic regression analysis was performed to examine the influence of sociodemographic, clinical, and psychological predictors on psychological distress (anxiety, depression, and somatization) before and after treatment, as assessed using the BSI-18 scale. Covariables included sociodemographic variables (such as sex, and age), clinical variables (performance status measured with the Eastern Cooperative Oncology Group scale (ECOG), cancer location and stage), and psychological variables (coping and spiritual well-being). Cancer localization was re-categorized into *k-1* dummy variables, where *bronchopulmonary* was the reference groups. SEM was used to identify the relationship between pre- and post-treatment psychological distress and the various coping strategies^26^. For all analyses, a significance level of α < 0.05 was adopted. Statistical analysis was performed using IBM SPSS Statistics for Windows, version 23.0 (IBM Corp., Armonk, N.Y., USA).

### Ethics approval and consent to participate

This study was approved by the Research Ethics Committee of the Principality of Asturias (May 17, 2019) and by the AEMPS (May 8, 2019). The studies have been performed in accordance with the ethical standards of the 1964 Declaration of Helsinki and its later amendments. This study is a prospective, observational, non-interventionist trial. Signed informed consent was obtained from all patients.

## Results

### Sociodemographic and clinical features

Between 2019 and 2022, 1977 patients were recruited, of which 1807 were eligible and 170 were excluded. Of those excluded, 40 did not meet any inclusion criteria, 42 met any exclusion criteria, and 88 had incomplete data at the time of analysis. Table 1 provides the baseline socio-demographic and clinical characteristics. A roughly equal proportion of men (46%) and women (54%) were included, with an average age of 64 years and 57% of participants being ≤ 65 years. Many participants were married or living with a partner (72%), had basic education levels (51%), and were either unemployed or retired (59%). The most common primary cancers were colorectal (30%), breast (25%), bronchopulmonary (18%), and non-colorectal digestive neoplasms (15%). Of the 944 participants with resected localized cancer, 19% were stage I, 36% were stage II, and 45% were stage III. Of the 863 participants with unresectable advanced cancer, 20% were stage III and 80% were stage IV.

**Table 1:** Demographic and clinical characteristics of patients (n = 1807) with corresponding *p*-values from analysis of variance (ANOVA) assessing psychological Distress (Anxiety, Depression, and Somatization) at treatment onset.

Demographic and clinical characteristics	N (%) (n = 1807)	Anxiety (mean ± SD)	Depression (mean ± SD)	Somatization (mean ± SD)
Sex				
Men	843 (46)	63.3 (7.9)	61.3 (6.5)	62.6 (7.8)
Women	964 (54)	63.9 (7.8)	62.1 (6.4)	63.2 (7.6)
* p* value		**0.001**	**0.003**	0.107
Age (years)				
≤ 65	1035 (57)	63.7 (7.9)	62.0 (6.4)	63.2 (7.7)
> 65	772 (43)	62.5 (7.9)	61.5 (6.6)	62.5 (7.6)
* p* value		0.002	0.088	0.044
Marital status				
Married/partnered	1301 (72)	63.1 (7.9)	61.5 (6.3)	62.7 (7.6)
No partnered	506 (28)	63.3 (7.9)	62.4 (6.8)	63.3 (7.8)
* p* value		0.697	**0.006**	0.163
Educational level				
Basic	919 (51)	63.3 (7.9)	61.7 (6.5)	62.7 (7.8)
Intermediate	888 (49)	63.1 (7.8)	61.8 (6.5)	63.1 (7.4)
* p* value		0.612	0.786	0.299
Employment status				
Employed	849 (47)	63.8 (7.9)	62.1 (6.5)	63.5 (7.7)
Retired or unemployed	958 (59)	62.6 (7.9)	61.4 (6.4)	62.3 (7.6)
* p* value		**0.002**	**0.001**	**0.001**
Cancer				
Colorectal	534 (30)	61.6 (7.8)	60.1 (6.1)	60.7 (7.1)
Breast	458 (25)	63.8 (8.0)	62.1 (6.3)	62.8 (7.3)
Bronchopulmonary	320 (18)	63.8 (8.0)	62.3 (6.8)	64.5 (8.1)
Digestive no colorectal	268 (15)	64.3 (7.9)	63.2 (6.2)	64.7 (7.3)
Others	227 (13)	63.2 (7.9)	62.6 (6.6)	64.1 (6.6)
* p* value		0.001	0.001	0.001
Stage				
Localized resected	944 (52)	62.1 (7.7)	60.6 (5.9)	61.1 (7.0)
Advanced unresecable	863 (48)	64.4 (7.9)	62.9 (6.9)	64.9 (7.8)
* p* value		**0.001**	**0.001**	**0.001**
Systemic treatment				
Chemotherapy (CT)	1087 (60)	62.6 (7.9)	61.4 (6.5)	62.3 (7.7)
CT and radiotherapy	312 (17)	63.0 (7.7)	61.6 (5.8)	62.1 (7.0)
Immunotherapy +- CT	62 (34)	65.7 (8.1)	63.2 (7.6)	65.3 (8.9)
Targeted therapy +- CT	46 (3)	62.7 (7.4)	61.8 (6.4)	65.0 (7.0)
Others	300 (17)	64.9 (7.8)	62.8 (6.8)	64.9 (7.6)
* p* value		**0.001**	**0.005**	**0.001**
Death				
No	1721 (95)	63.2 (7.9)	61.7 (6.5)	62.8 (7.6)
Yes	86 (5)	63.4 (7.6)	62.0 (6.9)	63.8 (8.6)
* p* value		0.822	0.712	0.272

*n* number of cases, *SD* standard deviation.

P-values deemed significant are highlighted in bold.

Table 1 summarizes the adjuvant treatments administered to patients with resected cancer, and the first-line treatments given to those with unresectable advanced cancer. Chemotherapy was the systemic treatment given to patients with resected cancer (100%), and radiotherapy was also included in 33% of cases. For those with unresectable advanced cancer, the most common therapy was chemotherapy (55%), and 34% received immunotherapy or 11% were treated with targeted therapy either alone or in combination with chemotherapy. Prior to the completion of adjuvant treatment (6 months) in patients with resected cancer, and prior to the first response evaluation study (2–3 months) in those with advanced cancer, 5% (n = 86) had passed away, with a greater proportion of deaths seen in patients with advanced cancer (17.8%) than those with localized cancer (3.3%).

Based on the BSI score, it was observed that 57% had scores indicating of anxiety, 44% indicating depression and 48% indicating somatization. As illustrated in Table 1, women were found to have higher levels of anxiety (*p* = 0.001) and depression (*p* = 0.003) than men, and those ≤ 65 years had higher levels of anxiety (*p* = 0.002) and somatization (*p* = 0.044) than the elderly. Additionally, patients without a partner displayed more depression than those with a partner (*p* = 0.006), and those in employment showed higher levels of anxiety, depression, and somatization than non-working patients (*p* = 0.002, *p* = 0.001, and *p* = 0.001, respectively). Patients with colorectal cancer were found to have the lowest levels of anxiety, depression and somatization compared to all other neoplasms (all *p* = 0.001). Furthermore, patients with advanced unresectable cancer had higher levels of anxiety, depression, and somatization than those with resected stage I–III cancer (all *p* = 0.001). Lastly, patients who only received chemotherapy (n = 1087) had significantly lower levels of anxiety, depression and somatization when compared to other groups (*p* = 0.001, *p* = 0.005, *p* = 0.001, respectively).

### Correlations across psychological variables and age

Table 2 presents the means, standard deviations, and Pearson correlation analyses of the psychological variables and age. The mean scores of anxiety, depression, and somatization were 63.2, 61.7, and 62.9, respectively. The two most employed coping strategies were positive attitude and cognitive avoidance, with respective mean scores of 77.3 and 59.3, whilst anxious preoccupation and hopelessness were the least utilized (33.4 and 33.8, respectively). Furthermore, the mean score of the FACIT-Sp spirituality scale was 34.7. The results demonstrated that there were significant correlations between all psychological variables apart from helplessness and positive attitude. It was found that positive attitude-based coping was associated with lower levels of anxiety (*r* = − 0.142, *p* < 0.001), depression (*r* = − 0.231, *p* < 0.001), somatization (*r* = − 0.087, *p* < 0.001) and anxious preoccupation (*r* = − 0.182, *p* < 0.001). Similarly, spirituality was linked to decreased anxiety (*r* = − 0.240, *p* < 0.001), depression (*r* = − 0.342, *p* < 0.001), somatization (*r* = − 0.148, *p* < 0.001) and anxious preoccupation (*r* = − 0.295, *p* < 0.001). Additionally, older age was associated with lower levels of anxiety (*r* = − 0.074, *p* < 0.001) and anxious preoccupation (*r* = − 0.103, *p* < 0.001), as well as higher levels of positive attitude (*r* = 0.046, *p* < 0.001), cognitive avoidance (*r* = 0.107, *p* < 0.001), and spirituality (*r* = 0.263, *p* < 0.001).

**Table 2:** Pearson’s correlations across psychological variables and age.

Variables	Mean ± SD	1	2	3	4	5	6	7	8	9
BSI. Anxiety	63.2 ± 7.9	1								
BSI. Depression	61.7 ± 6.5	0.763**	1							
BSI. Somatization	62.9 ± 7.6	0.514**	0.550**	1						
MAC: Helplessness	33.8 ± 25.3	0.385**	0.426**	0.300**	1					
MAC:Anxious preoccupation	33.4 ± 24.3	0.439**	0.441**	0.177**	0.238**	1				
MAC: Positive attitude	77.3 ± 16.7	− 0.142**	− 0.231**	− 0.087**	0.040	− 0.182**	1			
MAC: Cognitive avoidance	59.3 ± 26.3	0.190**	0.138**	0.100**	0.395**	0.168**	0.426**	1		
FACIT: Spiritual well-being	34.7 ± 7.6	− 0.240**	− 0.342**	− 0.148**	0.039**	− 0.295**	0.487**	0.178**	1	
Age	62.1 ± 11.9	− 0.074**	− 0.035	− 0.006	0.212**	− 0.103**	0.046*	0.107**	0.263**	1

*SD* Standard Deviation, *BSI* Brief Symptom Inventory, *MAC* Mental Adjustment Cancer, *FACIT* Functional Assessment of Chronic Illness Therapy-Spiritual Well-Being.

*p < 0.05 (two-tailed); **p < 0.01 (two-tailed).

### Changes in symptoms of anxiety, depression, and somatization before and after treatment

The Table 3 categorized the sample into four groups: patients who did not exhibit symptoms of anxiety, depression, or somatization either before or after treatment (never), those who did not show symptoms before treatment but did afterwards (post-treatment symptoms), those who initially showed symptoms but not afterwards (pre-treatment symptoms), and finally, those who exhibited symptoms both before and after treatment (persistent symptoms).

**Table 3:** Categorization of the sample into four symptom groups based on the presence of anxiety, depression, and somatization: pre-treatment, post-treatment, persistent (both pre- and post-treatment), or never.

Symptoms	Never n (%)	Pre-treatment n (%)	Persistent n (%)	Post-treatment n (%)
Anxiety	310 (32)	218 (22)	316 (33)	130 (13)
Depression	438 (45)	170 (18)	237 (24)	129 (13)
Somatization	296 (30)	128 (13)	309 (32)	241 (25)

Regarding anxiety, 22% exhibited high levels prior to the initiation of treatment, 33% had baseline anxiety that persisted during treatment, and 13% developed anxiety after 6 months of treatment. As for depression, 18% had baseline depression, 24% exhibited persistent baseline depression throughout the treatment, and 13% developed depression post-treatment. In the case of somatization, 13% had symptoms before starting treatment, 32% continued to experience symptoms after treatment, and 25% developed somatization symptoms after 6 months.

### Sociodemographic and clinical risk factors for anxiety, depression, and somatization

Multivariable logistic regression assessing the association between pre-treatment psychological distress and sociodemographic, clinical, and psychological variables is shown in Table 3. Higher levels of anxiety and depression were identified in individuals who reported using helplessness (OR 1.02, 95% CI 1.01–1.02 and OR 1.02, 95% CI 1.01–1.02, respectively) and anxious preoccupation-based coping (OR 1.04, 95% CI 1.03–1.04 and OR 1.04, 95% CI 1.03–1.05, respectively). Additionally, cognitive avoidance was associated with greater anxiety (OR 1.01, 95% CI 1.00–1.01). In contrast, lower levels of anxiety and depression were observed in older patients (OR 0.99, 95% CI 0.98–1.00 for both), men (OR 0.66, 95% CI 0.52–0.84 and OR 0.72, 95% CI 0.56–0. 93, respectively), those with a localized cancer (OR 0.39, 95% CI 0.27–0.55 and OR 0.28, 95% CI 0.19–0.41, respectively), and those with spiritual well-being (OR 0.97, 95% CI 0.95–0.98 y OR 0.93, 95% CI 0.91–0.94, respectively). In addition, those with a positive attitude demonstrated lower level of anxiety (OR 0.99, 95% CI 0.98–1.00). Those with colorectal cancer exhibited lower level of anxiety (OR 0.63, 95% CI 0.43–0.92) and depression (OR 0.55, 95% CI 0.37–0.81). Furthermore, somatization was observed to be higher in those with anxious preoccupation (OR 1.02, 95% CI 1.01–1.02), while older age (OR 0.99, 95% CI 0.98–1.00), colorectal cancer (OR 0.59, 95% CI 0.42–0.83), and localized (OR 0.40, 95% CI 0.29–0.56) had lower levels, as did those with spiritual well-being (OR 0.97, 95% CI 0.95–0.98) (Table 4).

**Table 4:** Multivariate logistic regression of sociodemographic and clinic variables correlated with psychological distress (anxiety, depression, and somatization) pre-treatment.

Variable	Anxiety					Depression					Somatization				
	95% CI					95% CI					95% CI				
	β	Wald test (z-ratio)	Odds ratio	Lower	Higher	β	Wald test (z-ratio)	Odds ratio	Lower	Higher	β	Wald test (z-ratio)	Odds ratio	Lower	Higher
Age	− 0.013	**6.290**	0.987	0.978	0.997	− 0.015	**7.896**	0.985	0.975	0.996	− 0.014	**8.749**	0.987	0.978	0.995
Sex: Male	− 0.417	**11.294**	0.659	0.517	0.840	− 0.332	**6.579**	0.717	0.557	0.925	− 0.221	3.795	0.801	0.641	1.001
ECOG: 0–1	0.071	0.063	1.074	0.616	1.873	− 0.246	0.709	0.782	0.441	1.387	− 0.112	0.190	0.894	0.541	1.479
Site: Bronchopulmonary	− 0.318	2.411	0.727	0.487	1.087	− 0.308	2.205	0.735	0.490	1.104	0.078	0.178	1.081	0.753	1.553
Site: Colorectal	− 0.458	**5.664**	0.632	0.434	0.922	− 0.605	**9.166**	0.546	0.369	0.808	− 0.532	**9.115**	0.588	0.416	0.830
Site: Digestive no colorectal	− 0.363	2.956	0.696	0.460	1.052	− 0.065	0.094	0.937	0.617	1.422	− 0.045	0.056	0.956	0.658	1.388
Site: Breast	− 0.185	0.792	0.831	0.553	1.249	− 0.224	1.109	0.799	0.527	1.213	− 0.155	0.683	0.856	0.593	1.237
Stage: Localized	− 0.949	**27.759**	0.387	0.272	0.551	− 1.260	**42.579**	0.284	0.194	0.414	− 0.908	**30.070**	0.403	0.291	0.558
MAC: Helpleness	0.015	**21.309**	1.015	1.009	1.022	0.016	**22.264**	1.016	1.009	1.023	0.004	1.486	1.004	0.998	1.010
MAC:Anxious Preoccupation	0.035	**116.643**	1.035	1.029	1.042	0.039	**144.201**	1.040	1.033	1.046	0.015	**30.737**	1.015	1.010	1.021
MAC: Positive Attitude	− 0.008	3.310	0.992	0.984	1.001	− 0.008	3.175	0.992	0.984	1.001	0.000	0.006	1.000	0.992	1.007
MAC: Cognitive Avoidance	0.007	**6.945**	1.007	1.002	1.012	0.001	0.142	1.001	0.996	1.007	0.002	0.566	1.002	0.997	1.007
FACIT: Spiritual well-being	− 0.033	**14.705**	0.967	0.951	0.984	− 0.078	**69.411**	0.925	0.908	0.942	− 0.035	**19.005**	0.966	0.951	0.981
Intercept	1.775	**9.722**	5.897			3.394	**32.380**	29.789			2.137	**17.030**	8.477		

*ECOG* Eastern Cooperative Oncology Group scale, *BSI* Brief Symptom Inventory, *MAC* Mental Adjustment Cancer, *FACIT* Functional Assessment of Chronic Illness Therapy-Spiritual Well-Being.

*Adjusted for demographic and clinical variables (sex, age, tumor site, tumor stage, and ECOG performance status).

Bold values indicate the significant at 5% level.

In the post-treatment phase, it was observed that 46% of patients displayed scores indicative of anxiety, 38% showed scores suggestive of depression, and 57% had scores consistent with somatization. Multivariable logistic regression assessing the association between post-treatment psychological distress and sociodemographic, clinical, and psychological variables is shown in Table 5. Anxious preoccupation was associated with increased risk of post-treatment anxiety (OR 1.02, 95% CI 1.01–1.03), depression (OR 1.02, 95% CI 1.01–1.03) and somatization (OR 1.01, 95% CI 1.00–1.02). In addition, higher levels of baseline anxiety (OR 2.16, 95% CI 1.59–2.93), depression (OR 3.15, 95% CI 2.26–4.39) and somatization (OR 2.78, 95% CI 2.08–3.72) were found to be associated with higher post-treatment psychological distress. Moreover, patients with a localized cancer were observed to have lower levels of anxiety (OR 0.44, 95% CI 0.28–0.71) and somatization (OR 0.62, 95% CI 0.39–0.98). Helplessness was also linked to a greater risk of somatization (OR 0.99, 95% CI 0.98–1.00).

**Table 5:** Multivariate logistic regression of sociodemographic and clinic variables correlated with psychological distress (anxiety, depression, and somatization) post-treatment.

Variable	Anxiety					Depression					Somatization				
	95% CI					95% CI					95% CI				
	β	Wald test (z-ratio)	Odds ratio	Lower	Higher	β	Wald test (z-ratio)	Odds ratio	Lower	Higher	β	Wald test (z-ratio)	Odds ratio	Lower	Higher
Age	− 0.003	0.186	0.997	0.984	1.010	0.004	0.375	1.004	0.991	1.018	− 0.008	1.624	0.992	0.979	1.004
Sex: Male	− 0.218	1.779	0.804	0.584	1.108	− 0.052	0.095	0.949	0.681	1.322	− 0.135	0.726	0.874	0.641	1.192
ECOG: 0–1	− 0.363	0.942	0.696	0.335	1.447	− 0.548	2.073	0.578	0.274	1.219	0.106	0.086	1.112	0.546	2.266
Site: Bronchopulmonary	− 0.231	0.642	0.794	0.452	1.396	− 0.146	0.243	0.864	0.483	1.546	− 0.233	0.658	0.792	0.451	1.391
Site: Colorectal	− 0.358	2.025	0.699	0.427	1.144	− 0.325	1.572	0.723	0.435	1.201	− 0.137	0.302	0.872	0.535	1.421
Site: Digestive no colorectal	− 0.274	0.903	0.760	0.431	1.339	− 0.540	3.255	0.583	0.324	1.048	− 0.251	0.768	0.778	0.444	1.364
Site: Breast	− 0.167	0.389	0.846	0.501	1.429	− 0.352	1.632	0.703	0.410	1.207	0.062	0.054	1.064	0.631	1.792
Stage: Localized	− 0.815	**11.357**	0.443	0.276	0.711	− 0.276	1.205	0.759	0.464	1.242	− 0.477	**4.155**	0.620	0.392	0.982
MAC: Helpleness	− 0.007	3.095	0.993	0.984	1.001	− 0.003	0.351	0.997	0.989	1.006	− 0.012	**8.671**	0.988	0.980	0.996
MAC:Anxious Preoccupation	0.022	**30.936**	1.022	1.014	1.030	0.017	**18.346**	1.018	1.009	1.026	0.010	**6.629**	1.010	1.002	1.017
MAC: Positive Attitude	0.000	0.000	1.000	0.989	1.011	− 0.002	0.197	0.998	0.987	1.009	0.002	0.207	1.002	0.992	1.013
MAC: Cognitive Avoidance	0.001	0.170	1.001	0.995	1.008	− 0.004	1.152	0.996	0.990	1.003	0.001	0.184	1.001	0.995	1.008
FACIT: Spiritual well-being	− 0.018	2.672	0.982	0.961	1.004	− 0.016	1.882	0.984	0.962	1.007	− 0.012	1.234	0.988	0.967	1.009
Score pretreatment	0.770	**24.282**	2.159	1.590	2.933	1.148	**46.118**	3.153	2.264	4.392	1.023	**47.418**	2.782	2.079	3.722
Intercept	0.766	1.050	2.150			0.128	0.027	1.136			0.894	1.514	2.445		

*ECOG* Eastern Cooperative Oncology Group scale, *BSI* Brief Symptom Inventory, *MAC* Mental Adjustment Cancer, *FACIT* Functional Assessment of Chronic Illness Therapy-Spiritual Well-Being.

*Adjusted for demographic and clinical variables (sex, age, tumor site, tumor stage, and ECOG performance status).

Bold values indicate the significant at 5% level.

### Relationship between pre- and post-treatment psychological distress and coping strategies, and path Analysis

**Figure 1:**
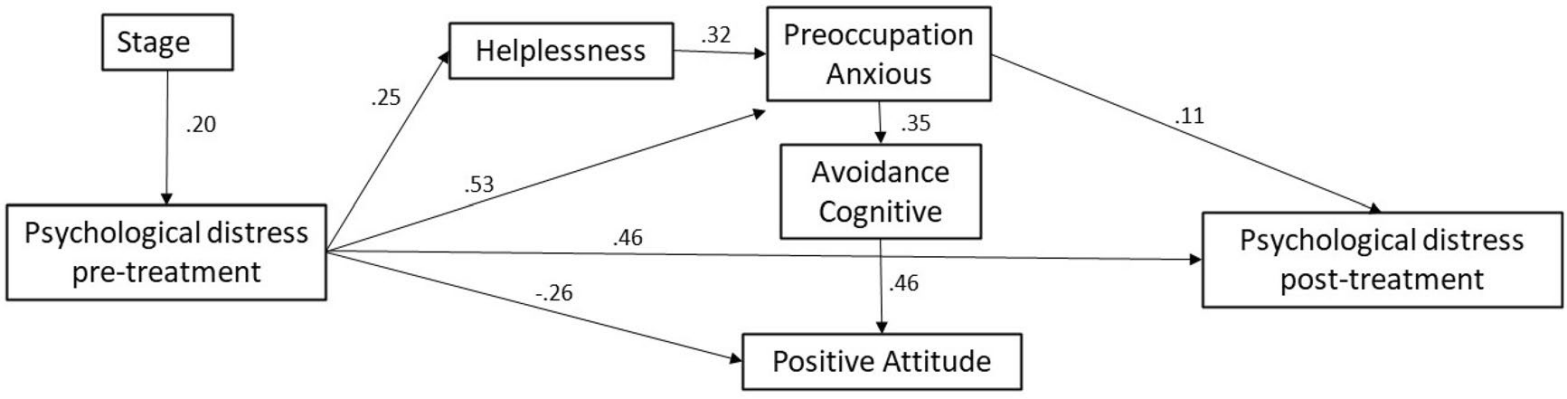
Path model and standardized factor weight of psychological distress post-treatment in the study. *Note* Standardized coefficients are presented, and all paths are significant at the 0.001 level. Chi-square = 27,255; probability level = 0.001; TLI = 0.953; CFI = 0.978; RMSEA = 0.067; NFI = 0.973.

The model exhibited good fit to the data, as indicated by the following statistics: χ2 = 27.255, *p* = 0.001; CFI = 0.978; TLI = 0.953; RMSEA = 0.067. As depicted in Fig. 1, pre-treatment psychological distress correlates positively with helplessness (β = 0.25) and anxious preoccupation (β = 0.53), and also influences post-treatment psychological distress (β = 0.46). Conversely, it shows a negative correlation with positive attitude (β = − 0.26). Furthermore, anxious preoccupation directly and positively impacts post-treatment psychological distress (β = 0.11).

## Discussion

This study found a correlation between psychological distress (including anxiety, depression, and somatization), younger age, and anxious preoccupation-based coping in a large sample of cancer patients (n = 1807). Conversely, localized resected cancer and spirituality were found to be protective factors. Male patients and those with a positive attitude were less likely to present with anxiety (OR 0.66, 95% CI 0.52–0.84 and OR 0.99, 95% CI 0.98–0.99, respectively) and depression (OR 0.72, 95% CI 0.56–0.92 and OR 0.99, 95% CI 0.98–0.99, respectively). Those with colorectal cancer were less prone to suffer from anxiety (OR 0.63, 95% CI 0.43–0.92), depression (OR 0. 55, 95% CI 0.37–0.81) and somatization (OR 0.59, 95% CI 0.42–0.83). After systemic cancer treatment, it was observed that patients with pre-existing anxiety (OR 2.16, 95% CI 1.59–2.93), depression (OR 3.15, 95% CI 2.26–4.39), and somatization (OR 2.78, 95% CI 2.08–3.72) experienced an increase in psychological distress. Similarly, those with anxious preoccupation developed an increased in anxiety (OR 1.02, 95% CI 1.01–1.03), depression (OR 1.02, 95% CI 1.01–1.03) and somatization (OR 1.01, 95% CI 1.00–1.02). Moreover, the path analysis reaffirmed that anxious preoccupation is associated with post-treatment psychological distress. On the other hand, those with localized cancer showed reduced levels of anxiety (OR 0.44, 95% CI 0.28–0.71) and somatization (OR 0.62, 95% CI 0.39–0.98).

We have yet to uncover prospective studies assessing the impact of sociodemographic, clinical, coping and spirituality variables on psychological distress in cancer patients, though studies analyzing the influence of several of these variables have been identified. Concerning sociodemographic factors, the available data suggests that gender and age influence psychological distress in cancer patients. A Chinese study conducted in patients with thyroid cancer, for example, reported that gender could be a predictor of psychological distress^27^. Additionally, a Turkish study showed that female gender was associated with a higher level of anxiety and depression in outpatients with cancer^28^, while a Spanish study that focused on patients with resected localized cancer noted that females were more likely to suffer from depression^10^. In line with this, another study from Spain demonstrated that male patients develop greater stoicism, exhibiting a higher tolerance to psychological suffering^29^. This finding may explain the reduced access to psychosocial support systems observed in male oncology patients^30^.

In terms of age, several publications have demonstrated that younger age can be a predictor of psychological distress. For instance, a study of patients with localized prostate cancer reported that younger age was associated with poorer psychological functioning^31^, while a study of breast cancer patients showed that younger age predicted greater psychological distress^32^. Similarly, an American study looking at the role of younger age in psychological distress found that younger age also predicted greater psychological distress^33^. This greater psychological distress found in younger patients may be associated with having cancer at a time of personal, family, and professional development when the disease can interfere with and compromise responsibilities ^34^.

To the best of our knowledge, no studies have examined tumor location as a predictor of psychological distress. However, some studies have evaluated psychological distress in patients with different tumor sites. Our group previously investigated the biopsychosocial and clinical characteristics of patients with resected localized colon and breast cancer and observed that patients with breast cancer had higher levels of anxiety, depression, and somatization before the start of adjuvant treatment. These findings may be attributed to the psychological distress caused by the body image impact of surgical treatment^35^. Interestingly, the differences are lost after adjuvant treatment is completed, possibly due to the adverse effects of systemic cancer treatment toxicity^36^. In the present study, we found that colon cancer is associated with lower baseline anxiety (OR 0.63, 95% CI 0.43–0.92), depression (OR 0.55, 95% CI 0.37–0.81) and somatization (OR 0.59, 95% CI 0.42–0.83)**.** However, this effect is again lost in the analyses performed at the end of treatment, probably due to the toxicity accumulated by cancer treatments, which is associated with an increase in psychological distress and affects all patients equally, regardless of tumor location. A study analyzing the spectrum of psychological disorders in cancer patients found that the greatest anxiety was suffered by patients receiving chemotherapy, a higher level of somatization was for those receiving both chemotherapy and associated radiotherapy, and depression affected more patients receiving only radiotherapy^37^. Our study also observed that patients receiving chemotherapy were the group with the lowest level of psychological distress compared to patients receiving other systemic cancer treatments (immunotherapy or targeted therapy). In future studies, it would be valuable to consider the primary tumor location, tumor extension, and expected response to systemic antineoplastic treatment. Understanding how these factors might influence prognosis and coping styles can provide a deeper insight into the psychological dynamics experienced by cancer patients.

The relationship between different coping strategies and psychological distress has been studied in several contexts. A study of patients with esophageal cancer found that anxious preoccupation and fighting spirit were strongly associated with psychological distress before surgical treatment, while after treatment, helplessness was the most linked coping strategy^38^. In a European study of patients with nasopharyngeal cancer, those with higher levels of anxiety and depression were more likely to use dysfunctional coping strategies such as helplessness and anxious preoccupation^39^. Similarly, cancer patients with an optimistic outlook had the fewest symptoms of anxiety and depression in other study^40^. It should be noted that most of these studies analyze the impact of coping strategies on psychological distress using a linear regression model, unlike our study which uses a multivariate model controlling for confounding factors. We found that anxious preoccupation was associated with anxiety, depression and somatization, helplessness with anxiety and depression, and cognitive avoidance with anxiety, while a positive attitude had a protective effect against anxiety and depression. Although the Mini-MAC scale might lead one to believe that cognitive avoidance could be adaptive, our findings have revealed an unexpected correlation with increased anxiety, suggesting a more complex relationship. Cognitive avoidance, driven by a persistent perception of threat and continuous efforts to evade cancer-related thoughts, may contribute to heightened anxiety rather than adaptive coping. Its effectiveness in the short-term contrasts with long-term ineffectiveness in managing cancer-related stress. Furthermore, our use of SEM revealed a significant relationship between psychological distress and coping strategies in patients with advanced cancer, highlighting the intensification of post-treatment psychological distress by anxious preoccupation. These findings align with those of other researchers who have observed an increase in psychological distress in patients following various oncological treatments. For instance, a study focused on quality of life in cancer patients identified a significant rise in psychological distress following chemotherapy^41^. Similarly, another study in breast cancer patients documented an increase in anxiety and depression post-treatment^42^. Our study builds upon these observations, demonstrating that there is an escalation of psychological distress after treatment connected to baseline anxious preoccupation. Additionally, a Japanese study also found that patients who express negative emotions experience greater psychological distress following surgery^43^. An American study identified a correlation between emotional and financial distress, attributable to the costs associated with cancer treatments^44^. The financial burden of treatments and the loss of productivity, owing to reduced patient functionality post-treatment, may further contribute to this increased psychological distress. Consequently, we believe it is essential to implement psychological interventions to improve coping strategies and mitigate the impact of psychological distress, especially post-treatment, thereby enhancing overall patient outcomes.

Spirituality has been found to predict psychological distress in cancer patients during the Covid-19 pandemic, with higher spirituality associated with lower distress. Previous research has corroborated our findings that spiritual well-being is associated with mental well-being and less anxiety, depression, and somatization^45,46^. Similarly, it has been observed that addressing spirituality appropriately can significantly influence positive patient outcomes during the oncological process^47^. Thus, other studies have demonstrated that cancer patients utilize spirituality as a coping mechanism during their illness, helping them to contend with experiences that threaten their sense of lifey^48^.

The current study has several limitations that should be noted. Firstly, although the effect of different coping strategies was statistically significant, it was minimal in a large sample size with sufficient power. Secondly, the definition of psychological distress, anxiety, depression, and somatization was based on the BSI-18 scale rather than a clinical diagnosis. Thirdly, post-treatment analyses were conducted at different times for patients with localized or advanced cancer, at 6 months and 2–3 months, respectively. This was due to the prolonged treatment of patients with advanced disease until progression or unacceptable toxicity, with the risk of increased losses if the assessment was prolonged. Fourthly, although the study was controlled for clinical, socio-demographic, and psychological variables, there may have been other factors that influenced the psychological distress of the patients that were not considered. Finally, the questionnaires used were self-completed, which may lead to response bias due to errors in interpretation, inaccurate recall, or difficulty in understanding them. Although these questionnaires have proven useful in assessing psychological distress, coping and spiritual wellbeing, they should ideally be used in conjunction with a clinical assessment.

In conclusion, this study has identified several sociodemographic, clinical, and psychological variables that may predict psychological distress in cancer patients. These include young age and female sex, the presence of advanced unresectable cancer, and cancer location outside the colon, as well as anxious preoccupation and lack of spirituality. Further research is needed to confirm these findings and inform effective interventions to address psychological distress in cancer patients.

## Data Availability

Statistical analyses were performed with Statistical Package for Social Sciences (SPSS) software, 25.0 version (IBM SPSS Statistics for Windows, Armonk, NY: IBM Corp). The code is available upon request to the authors.

## Code availability

Patients are identified by an encrypted code known only to the local researcher. The code of the analyses is available upon request to the authors.
